# The Environmental Impacts of Disposable Nonwoven Fabrics during the COVID-19 Pandemic: Case Study on the Francesc de Borja Hospital

**DOI:** 10.3390/polym15051130

**Published:** 2023-02-23

**Authors:** Alberto Quintana-Gallardo, Romina del Rey, Salvador González-Conca, Ignacio Guillén-Guillamón

**Affiliations:** 1 Center for Physics Technologies (CTFAMA), Universitat Politècnica de València, 46022 Valencia, Spain; 2 Center for Physics Technologies (CTFAMA), Escola Politècnica Superior de Gandia, Universitat Politècnica de València, 46730 Gandia, Spain; 3 Departament de Salut, Hospital Francesc de Borja, 46730 Gandia, Spain

**Keywords:** hospital waste, nonwoven fabrics, plastic waste, carbon footprint, LCA, COVID-19

## Abstract

Hospitals generate huge amounts of nonwoven residues daily. This paper focused on studying the evolution of nonwoven waste generated in the Francesc de Borja Hospital, Spain, over the last few years and its relation to the COVID-19 pandemic. The main objective was to identify the most impacting pieces of nonwoven equipment in the hospital and to analyze possible solutions. The carbon footprint of the nonwoven equipment was studied through a life-cycle assessment. The results showed an apparent increase in the carbon footprint in the hospital from 2020. Additionally, due to the higher annual volume, the simple nonwoven gown used primarily for patients had a higher carbon footprint over a year than the more sophisticated surgical gowns. It can be concluded that developing a local circular economy strategy for medical equipment could be the solution to avoid the enormous waste generation and the carbon footprint of nonwoven production.

## 1. Introduction

Hospitals generate enormous amounts of residues every year. As a critical sector in society, the environmental impacts caused in hospitals are generally considered acceptable and necessary. In recent decades, there has been a worldwide increase in medical textile consumption [1]. According to Uddin and Chaudhary, this increase is driven by several factors: the rising population, the higher age average, the increased access to healthcare, and the rise in chronic diseases.

Amidst the COVID-19 pandemic, these impacts radically increased due to the higher usage of personal protective equipment (PPE) (A list of abbreviations can be found in Table S1) [2]. Public opinion and the media commonly focused their attention on protective masks. However, there are other kinds of medical equipment of which usage has increased since 2020. This is the case with medical gowns, traditionally made of cotton and washed in the laundry after every use. In fact, some decades ago, many big or even medium size hospitals in Spain had their laundry service in the building. In recent times, most hospitals decided to externalize this service [3].

Since the 1990s, there has been a global tendency of transitioning from cotton garments to single-use nonwoven ones [4]. Nonwoven, commonly called nonwoven fabrics, are materials made from plastic fibers that imitate the texture and look of conventional fabrics. These plastic fabrics are bonded either mechanically, chemically, with heat, or with some solvent. The most common polymers used to manufacture nonwovens are polypropylene (PP) and polyester [5]. There are many kinds of different processes used to bind the polymers together. SMS (spunbond/meltblown/spunbond), spunlace, and spundbound are the most frequent in the case of the medical industry. In the case of medical gowns, the production process is mainly carried out through a spunbond or an SMS process [6].

Even before the coronavirus pandemic of 2020, the simplicity, safety, and hygiene of single-use nonwovens had already convinced many medical industry professionals to use them despite their drawbacks in terms of waste generation. After the pandemic began, the benefits provided by nonwovens became more critical than ever before, as the safety measures to avoid infections were very strict. There was an increasing concern about the safety of medical personnel. This led to medical personnel leaning towards using disposable PPE. Their perception of disposable PPE was that they can guarantee the level of effectiveness stated in the technical documentation.

The effectiveness as a barrier of surgical gowns was classified by the Association for the Advancement of Medical Instrumentation in the ANSI/AAMI PB70:2012. This norm established a system of classification for protective apparel and drapes used in healthcare facilities based on their liquid barrier performance and specified related labeling requirements and standardized test methods for determining compliance [7]. As well as gowns, this norm also includes coveralls, aprons, drapes, and other medical protective clothing. Table 1 describes the four protection levels and the test required for the certification.

Nonwoven gowns have been shown to be able to perform up to the required medical standards. Kumar Midha et al. reviewed the protection of several nonwoven medical gowns. They observed that the SMS of 35 and 50 g/m^2^ weight offered sufficient liquid barrier properties for level two protection, as per the Association for the Advancement of Medical Instrumentation barrier protection classification, without applying fluorochemical and antibacterial finishes [6]. However, spunbond fabrics of 35 and 50 g/m^2^ weight offered only level one protection. However, a study highlighted that the elevated security of disposable garments might sometimes be false. McQuerry et al. analyzed the differences in performance between disposable and reusable medical garments in other studies [8]. They tested some of the performance parameters of several reusable and disposable gowns, such as average impact penetration and average hydrostatic pressure. Their findings determined that some of the disposable gowns available on the market did not meet the AAMI PB70 performance requirements for HCW protection. Moreover, the use of disposable gowns also has other drawbacks. Hicks et al. signaled the higher cost of single-use PPEs compared to reusable ones. Their study also quantified the amount of waste generated by using single-use PPEs. Moreover, it emphasized the risks associated with possible supply restrictions due to spikes in world demand [9]. Despite all of these potential drawbacks, the use of disposable nonwoven PPEs continues in an ascending trend, due primarily to their practicality, both for the logistic department in hospitals and the medical personnel.

Since 2020, several studies analyzed the environmental impacts related to the COVID-19 pandemic. Rume and Islam developed a holistic study on the global environmental effects of the pandemic. The study highlighted the overgeneration of waste due to the increase in the use of disposable PPE through a literature review [2]. Klemeš analyzed the present and future possibilities of reducing plastic waste related to COVID-19 [10]. The study analyzed the possibilities of avoiding the environmental impacts of disposable PPEs by creating reusing and recycling programs. Moreover, it highlighted the risk that outsourcing the production of these kinds of critical products poses for Western countries. Zhao et al. compared the differences in the environmental impacts of biodegradable disposable nonwoven gowns and conventional ones [11]. The study showed that biodegradable gowns, made from biodegradable PP, have higher environmental impacts in categories such as ecotoxicity (10.76%) and lower land use and greenhouse gas emissions (48.81% and 5.67, respectively). However, using biodegradable disposable gowns does not solve the possible lack of PPE in medical centers due to market chain disruptions or peaks in consumption. Another study by Zhao et al. analyzed the energy and environmental sustainability of waste personal protective equipment (PPE) treatment under COVID-19. In 2022, Corbin et al. audited a Neurosciences Intensive Care Unit in the US to analyze the possibilities of diverting waste generation from landfills. The study showed that 24.7% of the waste produced in that unit could be either composted, recycled, or even sterilized for further reuse [12]. In December 2022, Kheirabadi and Sheikhi conducted a scoping analysis of the risks, benefits, and opportunities that arose from recycling or reusing biomedical materials. The study highlighted the need to strictly follow safety and hygiene protocols to reduce the risk of contamination [13].

To our knowledge, there has not been a study that analyzed the life cycle of the annual disposable gown consumption in hospitals. Therefore, there are questions related to this issue that need to be answered. What are the most impacting gowns over the life cycle of the hospital? What are the bottlenecks towards achieving less environmentally impacting hospitals?

This study explored the evolution of single-used materials made of nonwoven fabrics over the last four years in the Francesc de Borja Hospital, located in Gandia, Valencia (Spain). The main objective was to analyze and compare the differences between the impacts related to the production process of the medical gowns used in that hospital and to study how those impacts translated on an annual basis.

## 2. Materials and Methods

### 2.1. Nonwoven Use in the Francesc de Borja Hospital

The Francesc de Borja Hospital is a public medical center in Gandia, a medium size town located in the Valencian Community (Spain). This hospital, inaugurated in 2015, provides medical assistance to more than 188,000 inhabitants of the 31 municipalities of the Safor region and the ten towns of La Vall d’Albaida [14]. The Francesc de Borja hospital in Gandia contains 292 hospital rooms. All of them are individual rooms, and 32% have the possibility of double occupancy in extraordinary cases, bringing the total number of beds to 388. To this must be added the 13 intensive care unit (ICU) and 10 neonatal unit beds, reaching a maximum of 411 beds. The hospital currently employs 1106 workers. 

There were five main kinds of nonwoven gowns used in the Francesc de Borja Hospital, each one for a different purpose:

- Non-sterile gown: this gown is made with a single layer of spunbond nonwoven polypropylene. It is used in non-surgical applications.
- Sleeveless gown: this gown shares all the characteristics of the non-sterile gown, with the only difference being that it is sleeveless.
- Sterile surgical gown: This gown is made with Spunbond, Meltblown, Meltblown, Meltblown, and Spunbond (SMMMS). This means that the fabric is composed of five layers, one spunbond nonwoven polypropylene on each side and three layers of meltblown nonwoven polypropylene in the middle. The highest level of the commonly seen gowns in the market is typically made from this material, which provides the highest level of protection against fluids. These are ANSI/AAMI Level 2 surgical gowns.
- High-risk sterile surgical gown: Similar to the sterile surgical gown, this gown is made using SMMMS. In this case, the difference is that the layers’ density is higher and thus reaches ANSI/AAMI Level 4.
- Reinforced sterile surgical gown: This gown is made using polyester + PE, which means that it is made with a spunbond nonwoven polyester layer, a reinforcing, and a waterproofing layer of polyethylene. These are ANSI/AAMI Level 3 gowns.

As mentioned in the introduction, there has been a significant increase in the use of nonwoven material after the pandemic. The evolution over the last four years can be seen in Table 2. The weight of the different gowns was obtained by measuring several units of each gown type. The scale used was the Precisa 620c. The scale had an accuracy of ±0.01 g.

The amount of residues increased proportionally with the amount of single-use products used. The waste needs to be classified according to stringent rules. The local legislation in the province of Valencia classifies sanitary waste into four categories depending on its characteristics and potential risks [15]. Each type has different waste processing needs and classification strategies:

- Type I. Conventional residues: While generated at the hospital, type I residues are not specific to the medical activity. This category includes cardboard, office supplies, food waste, or furniture. Those residues, which do not require any particular management practice, are classified in black bin bags.
- Type II. Non-specific sanitary residues: This type includes waste produced due to medical activity that has not been in contact with any infectious disease. Items such as casts, bandages, waste derived from small surgical interventions, or any other material not included in type III. Waste included in this group is classified in green bin bags.
- Type III. Special sanitary waste includes bio-contaminated materials, generally considered hazardous waste. This category includes materials in contact with infectious diseases, anatomic residues (not including corpses or corpse remains), blood and blood products in liquid form, needles, and other sharp materials and vaccines. This type of waste must be deposited inside red bags placed in rigid bins adequately identified with the official logo of bio-contaminated residues and the text “hazardous waste” written next to it.
- Type IV. Waste typified by specific regulations: This type includes cytostatic substances, traces of toxic or hazardous chemical substances, expired medical drugs, toxic metals, and radioactive residues. These residues must be placed in a single-use container with the external identification “Chemically contaminated material. Cytostatics”. Medical garments such as gowns have generally been considered type II residues. However, after the pandemic, every material was considered in contact with coronavirus and automatically classified as a type III residue.

### 2.2. Life Cycle Assessment Methodology

A comparative life-cycle assessment of each medical gown was conducted, following the guidelines of the ISO 14040 and the ISO 14025 [16,17]. The modules studied were A1 to A5, therefore taking into account every process from the extraction of the raw materials to the transportation to the hospital. The allocation principle used in this study was allocation at end-of-life (EoL) according to the ISO 14025. Simapro v9 was used to create the life-cycle inventory (LCI). Simapro incorporates Ecoinvent V3.8, the most comprehensive database for life-cycle assessment studies.

The calculation method used to obtain the results was the Environmental Footprint methodology v3, developed by the Joint Research Centre of the European Commission. Documentation of the method and its normalization and weighting process was developed by [18]. The European Commission recommends this methodology for studies in the European context [19].

As seen in Table 1, data from all the single-use materials used in the hospital were collected and studied to determine their composition, weight, country of origin, and other relevant data required for the LCA study. Each of the gowns came from a different manufacturer. The non-sterile and sleeveless gown only contained nonwoven PP and rubber bands on the wrists. In addition to the PP nonwoven and the rubber bands, the sterile surgical gowns included a hook and loop (commonly called Velcro) on the neck area. The reinforced sterile gown, size L, was made of three layers, one of polyester nonwoven, one of fleece made of polyethylene, and the reinforcing layer was made of polyethylene. The reinforced sterile gown size XL, provided by a different manufacturer, was made of nonwoven PP and two protective layers, one of a higher density nonwoven PP and one of polyethylene fleece. The high-risk sterile surgical gown was made of nonwoven PP and a protective layer of polyethylene terephthalate. The energy required to sew the gowns and the impacts related to the transportation from China to the hospital were also included in the life-cycle inventory (LCI).

## 3. Results

**Figure 1:**
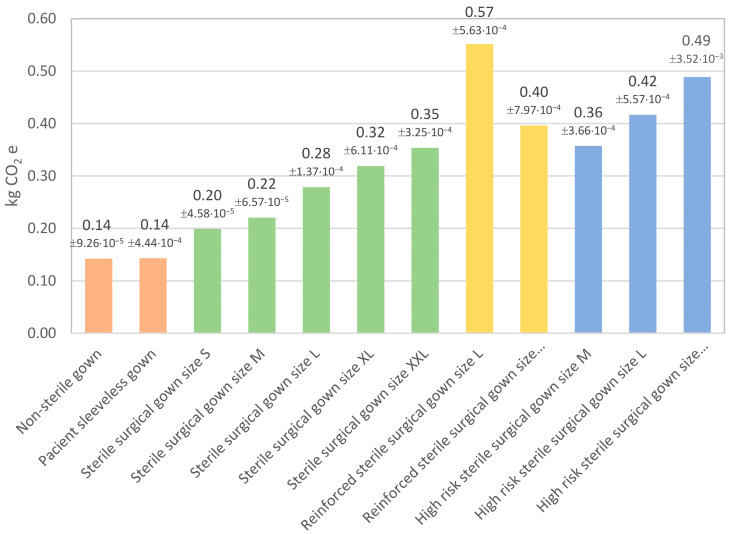
Carbon footprint of one gown unit.

Table 3 and Table 4 show the Environmental Footprint characterization results. The tables show the environmental impacts of the surgical gowns used in the Frances de Borja Hospital in fifteen categories. As expected, the results indicated that the more simple and lightweight gowns made lower environmental impacts in every category. The results were especially relevant in the case of climate change (Figure 1), in which the carbon footprint of a single gown ranged from 0.14 kg of CO_2_e, in the case of the simple non-sterile gown, to 0.55 in the reinforced sterile surgical gown. Generally, gowns with a higher quantity of material had a higher carbon footprint. However, the size L reinforced surgical gown had a higher carbon footprint due to the polyester layer. As it was explained in previous sections, the two reinforced gowns were produced by different manufacturers and slightly different materials.

Table 5 shows each material’s percentual contribution to each gown’s carbon footprint. The production of the nonwoven material was the most carbon-intensive process, even in the case of the reinforced sterile surgical gown, in which the fabric was composed of three layers. 

**Figure 2:**
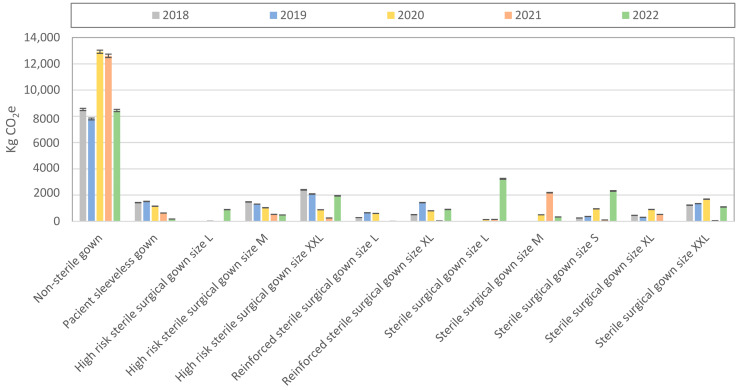
Annual carbon footprint of the total gown consumption per gown type.

**Figure 3:**
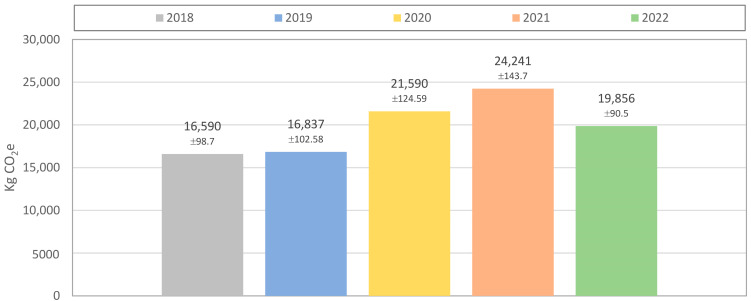
Annual carbon footprint of the total gown consumption.

Figure 2 shows the carbon emissions of each gown type over the time period of a year. Upon reviewing the annual CO_2_e emissions, the less impacting gown, per unit, was responsible for most of the total annual carbon footprint of the nonwoven gowns. Figure 3 depicts the annual carbon footprint of the total gown consumption. A decrease in carbon emissions in 2022, due to the attenuation of the COVID-19 pandemic crisis, was observed.

### 3.1. Preliminary Analysis of the Potential Benefits Associated with the Implementation of a Circularity Model

The results obtained in the previous categories indicate that the non-sterile gown had the highest environmental footprint, especially concerning the carbon footprint. This particular gown presented some characteristics that facilitated the design and implementation of a circular economy model. Due to the simplicity of its composition, recycling this gown does not require any kind of separation. That reduces the operational costs that manual separation would entail. Additionally, contrary to the more protective gowns, the non-sterile gown is never used in situations with the presence of infectious agents. This reduces the risk of contamination through the remanufacturing process.

**Figure 4:**
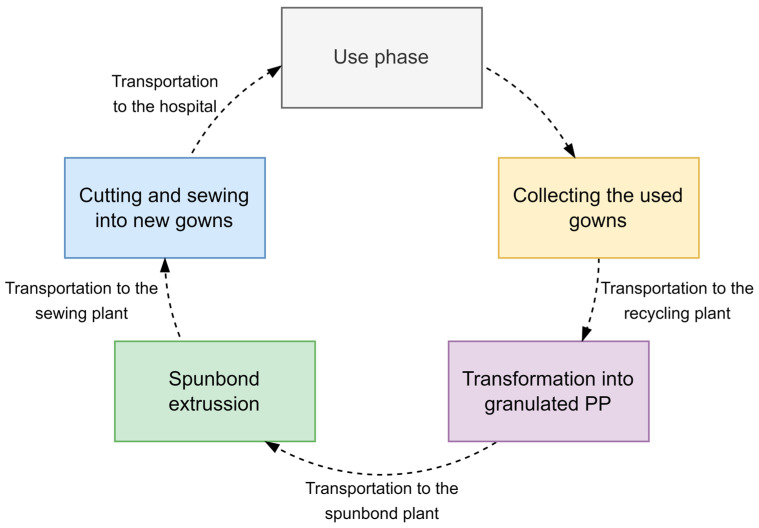
Preliminary design of the circular economy mode.

An outline of the potential circular economy model is depicted in Figure 4. After being used, the gowns would be collected and transported to a recycling plant. The used gowns would be washed, shredded at the recycling plant, and converted into granulated polypropylene. The granulated PP would then be transported to a plant equipped with a spunbond extruding machine, where it would be turned into nonwoven material again. At that point, the material would be ready to be cut and sewed into gowns again. The gowns would finally be transported back to the hospital, where the loop would start again.

The data for the carbon emissions associated with the reprocessing of the materials were obtained from Ecoinvent V3.8. The transportation between the different reprocessing facilities was assumed to be carried out using a seven-tonne lorry. The distances were estimated through an analysis of the plastic companies present in the area. The distance from the hospital to the nearest PP recycling plant was 50 km, 700 km from the recycling center to the spunbond plant, 700 km to the cutting and sewing company near the hospital, and 20 km from that company to the hospital again.

**Figure 5:**
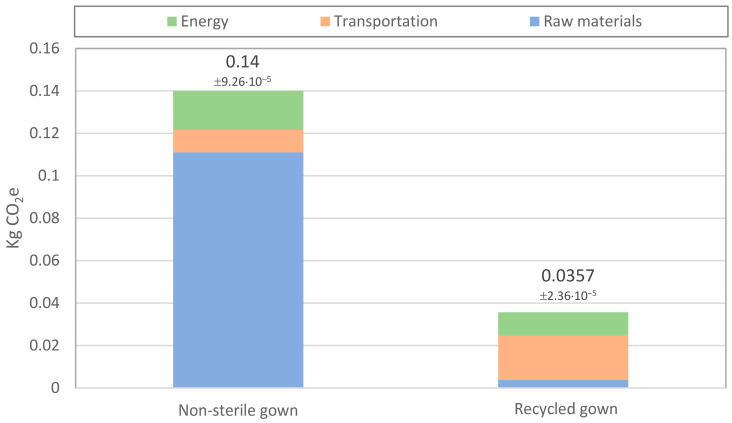
Comparison of the non-sterile gown and the recycled gown carbon emissions.

Figure 5 depicts the comparison between the carbon footprint of the conventional non-sterile gown and the potential results of a circular economy-based gown. The results showed that the recycled gown could potentially reduce carbon emissions by 75%. The results were divided into three categories: energy, transportation, and raw materials. Due to the reuse of the polypropylene contained in the used gowns, the carbon emissions associated with raw materials would be reduced to the minimum in the case of the recycled gown. This is also the case with the energy use, as well as with energy use, which would be around 30% lower. However, the carbon emissions associated with transportation would be increased. This is because the connection between the different manufacturing companies would be produced by road using trucks and also due to the distance (around 700 km) from the hospital to the spunbond extrusion manufacturing plant. If there was a plant with similar characteristics in the vicinity of the hospital, the carbon emissions would be further reduced.

## 4. Discussion

The use of nonwoven surgical gowns in the Francesc de Borja Hospital is a significant source of carbon emissions. Contrary to what might have been expected, the simpler gown was the one that contributed the most to the carbon footprint over a year due to the sheer amount being used. It should also be noted that, as well as the carbon emissions, the gowns lead to the annual generation of between 6800 and 10000 kg of plastic waste. 

During the year 2022, there was a slight decrease in the consumption of nonwoven fabrics compared to the years 2020 and 2021, which also led to a reduction in the carbon footprint of the Francesc de Borja Hospital. This was probably due to the decrease in coronavirus cases in the area. However, nonwoven fabric consumption has not returned to the levels prior to the pandemic. The use of disposable gowns in the hospital continues to be impactful on the environment.

Although single-use nonwoven gowns are not the best option in terms of sustainability, avoiding, or even banning, the use of nonwoven fabrics in hospitals is not possible due to the critical importance of maintaining adequate levels of hygiene, especially in the context of a pandemic. The costs associated with waste management also play a critical role in incorporating effective measures, as was analyzed in a comprehensive study on the Greek public health system [20]. Although biobased nonwovens are making their way in some industries, such as the agricultural sector, the strict quality control of the medical industry makes it harder to incorporate innovate bio-based solutions [21]. Biodegradable nonwovens do not seem to be the solution either, in terms of their environmental impacts during their production, according to a study by Zhao et al. [11]. The most plausible way to reduce the environmental impact of disposable nonwoven gowns is to create circular economy models. 

The circular economy consists of turning goods at the end of their service life into resources for others, closing loops, and minimizing waste [22]. In this case, this would involve converting the used nonwoven gowns into raw materials again, from which it could be possible to remanufacture gowns. Developing a local circular economy model could solve the two most important issues related to disposable nonwoven PPEs: their environmental impacts and their possible unavailability in cases of disruption of the supply chains or peaks in demand. A clinical trial of the University Health Network of Toronto highlighted the risks associated with the absence of sufficient personal protective equipment (PPE) in hospitals due to the disruption of supply chains and the necessity of having local PPE manufacturing [23]. In the case of the environmental impacts, as seen in Table 5, most of the carbon footprint of the gowns stemmed from the obtention and processing of the raw materials.

However, developing and putting into practice circular economy models can be challenging, for four primary reasons: medical waste regulations, coordination between companies, hospital logistics, and technical difficulties.

- Medical waste regulations: Regulations in some countries make it difficult to repurpose medical waste, as all the COVID-19-related waste in Valencian hospitals is currently classified as a type III residue. Type III residues must be burned through very strict procedures. This impedes the waste from being used in a circular economy model, as the disinfection of waste can be challenging [21].
- Hospital logistics: The hospital’s medical and logistics personnel must be committed to the circular economy model. Separating the different kinds of gowns is instrumental to developing a circular economy model in a medical context.
- Coordination between companies: To create a circular economy model, it would be necessary to coordinate a consortium of at least three companies. First, a company that collects the used gowns and transforms them into new raw materials, in the case of polypropylene, into PP granulate. Secondly, a company in charge of transforming the new raw material into nonwoven again. Finally, a company in charge of the cutting and sewing processes manufactures the new gown. Coordinating with several companies can be challenging.
- Technical difficulties: Gowns made from more than one kind of plastic cannot be directly introduced into the recycling machinery. Their materials must be separated for them to be recyclable. For example, in the case of the reinforced sterile surgical gown, the recycling process would need to start by separating the polyester, the fleece, and the PET. This is likely not realistic and would be labor-intensive. Even elastic rubber bands and hook-and-loop parts would need to be separated from the main nonwoven fabric. Not separating the materials would make it impossible for the spunbond extruder to function and could potentially damage the machine.

The difficulties associated with circularity in the medical industry have been the subject of many studies over the last few decades. Researchers have explored the potential of recycling medical plastic waste at least since the early 2000s, a decade before the European Commission launched its first action plan on the circular economy [24]. In 2002, Lee et al. analyzed the possibilities of recycling medical waste and highlighted the difficulties associated with the risk of infection [25]. 

In 2018, a review study by Kane et al. analyzed the state of the circular economy in the medical industry [26]. The study concluded that some circularity already existed in the medical industry, especially in complex and expensive medical equipment. However, the study highlighted the lack of development that the circular economy had in nonwovens and the opportunity it represented. As already highlighted by other studies, this study explained recovery’s dependence on hygienic criticality and infection control requirements. Additionally, in 2018, Voudrias highlighted the importance of approaching healthcare waste management from a circular economy perfective and listed six steps that any medical organization could apply to develop a circular economy: create a team of professionals in the hospital focused on circularity, measure waste production, minimize the production of waste, safe reuse of materials, and recycling [27]. That same year, the World Health Organization released a report analyzing the challenges and risks of reintroducing materials from medical waste again into the medical context, even after being reprocessed [28].

In 2019, Scavarda et al. published a study on the waste management system of Brazilian hospitals. Based on the triple bottom line, the study concluded that hospitals must develop educational programs, foster corporate responsibility, and interact with the community [29]. This requires a significant economic investment that not every country can afford. Following the lines of this study, in 2021, a review study of the consequences of inadequate healthcare waste management practices highlighted the differences between countries depending on their wealth [30]. Additionally, in 2021, a study showed the feasibility of reprocessing face masks and the environmental benefits that implementing those strategies would have [31]. 

After considering all the information collected in this study, the Francesc de Borja Hospital will develop a pilot circularity model in collaboration with local companies. After analyzing the results presented in this study, the hospital decided to use the non-sterile surgical gown as the basis for the pilot project. As explained in Section 3.1, there are three reasons that support this decision. First, as explained in the methodology section, the non-sterile surgical gown is only composed of a layer of nonwoven polypropylene. Because of the absence of rubber bands or hoop-and-loops in its design, it would be possible to introduce the gowns directly into the loop without the need for separation. Additionally, these gowns are rarely used when there is a risk of infection. That would reduce the possibility of spreading viruses through the circular chain. Moreover, the circular model will be designed to be handled only by local materials. A simpler gown such as this will make it easier for small local Spanish companies to carry out the cutting and sewing processes. Finally, the non-sterile surgical gown is the most impacting gown over a one-year period in the hospital. As shown in Section 3.1, implementing a circular economy model based on remanufacturing the non-sterile gowns from used gowns would imply reducing carbon emissions by 75%.

## 5. Conclusions

It is possible to draw several conclusions from this work:

- As was expected, the more sophisticated gowns were those with a higher environmental impact overall, especially the high risk sterile surgical gown;
- Despite the lower environmental impacts per unit in every environmental footprint category, the non-sterile gowns were the ones that contributed more to the impacts generated by the Francesc de Borja Hospital due to their high annual consumption;
- Due to the high annual consumption, the non-sterile gown had more than a 600% higher annual carbon footprint than any other gown in the hospital;
- Gowns that incorporated nonwoven polyester tended to have higher environmental impacts than those made from nonwoven polypropylene;
- The consumption of disposable nonwoven fabrics in the hospital decreased slightly in 2022. However, it has not returned to the levels of the years prior to the pandemic;
- Considering that the non-sterile gown was mostly made of PP, designing a circular economy model for it would be easier than doing so for other gowns. Moreover, it would be more effective in reducing the carbon footprint of the hospital due to the higher consumption of these gowns;
- A circular economy model based on the non-sterile gown could reduce carbon emissions by 75% compared to the conventional manufacturing process of the gown.

In future research, it will be possible to start working on the design of a circular economy model for the non-sterile gown. By doing so, it will be possible to avoid the emissions related to obtaining raw polypropylene granulate, which is one of the higher sources of carbon emissions in its manufacturing process. Additionally, if the circular economy model was designed to be implemented at a local level, it would be possible to reduce the impacts related to transportation. In that process, it would be necessary to find a polypropylene recycling plant that could turn the used gowns into polypropylene granulate again, and another manufacturing plant equipped with a spunbond extruder.

## Figures and Tables

**Table 1:** ANSI/AAMI PB 70:12 classification of barrier performance of surgical gowns [7].

Level ^1^	Test	Liquid Challenge	Result	Expected Barrier Effectiveness
1	AATCC 42 Impact Penetration ^2^	Water	4.5 g	Minimal water resistance (some resistance to water spray)
2	AATCC 42 Impact Penetration	Water	1.0 g	Low water resistance (resistant to water spray and some resistance to water penetration under constant contact with increasing pressure)
	AATCC 127 Hydrostatic Pressure ^3^	Water	20 cm	
3	AATCC 42 Impact Penetration	Water	1.0 g	Moderate water resistance (resistant to water spray and some resistance to water penetration under constant contact with increasing pressure)
	AATCC 127 Hydrostatic Pressure	Water	50 cm	
4	ASTM F1670 Synthetic Blood Penetration Test (for surgical drapes)	Surrogate Blood	no penetration at 2 psi (13.8 kPa)	Blood and viral penetration resistance (2 psi)
	ASTM F1671 Viral Penetration Test (for surgical and isolation gowns)	BacteriophagePhi-X174	no penetration at 2 psi (13.8 kPa)	

^1^ In order of increasing protection. ^2^ American Association of Textile Chemists and Colorists (AATCC) 42 Water resistance: impact penetration test determines the ability of a material to resist water penetration under spray impact [AATCC 2000]. ^3^ AATCC 127 Water resistance: hydrostatic pressure test determines the ability of a material to resist water penetration under constant contact with increasing pressure [AATCC 1998].

**Table 2:** Disposable nonwoven usage in the Francesc de Borja Hospital.

Item	2018		2019		2020		2021		2022	
	Units	kg	Units	kg	Units	kg	Units	kg	Units	kg
Non-sterile gown	59,860	3155.82 ± 14.37	54,793	2888.69 ± 13.15	90,777	4785.76 ± 21.79	98,741	5205.63 ± 23.7	59,335	3128.14 ± 14.24
Sleeveless gown	10,048	532.79 ± 11.50	10,621	563.18 ± 12.16	8015	424.99 ± 9.18	13,081	693.62 ± 14.98	1296	68.72 ± 1.48
High risk sterile surgical gown L	1098	157.79 ± 0.49	739	106.2 ± 0.33	2167	311.41 ± 0.96	1690	205.75 ± 0.75	2142	307.82 ± 0.95
High risk sterile surgical gown M	723	88.02 ± 0.24	1045	127.22 ± 0.35	2651	322.75 ± 0.89	1683	241.86 ± 0.56	1369	166.67 ± 0.46
High risk sterile surgical gown XXL	2506	427.67 ± 6.11	2760	471.02 ± 6.73	3460	590.48 ± 8.44	2825	482.11 ± 6.89	3984	679.91 ± 9.72
Reinforced sterile surgical gown L	0	0	0	0	256	34.17 ± 0.08	3654	487.74 ± 0.21	19	2.54 ± 0.01
Reinforced sterile surgical gown XL	0	0	0	0	1264	179.44 ± 0.9	722	102.5 ± 2.59	2286	324.52 ± 1.62
Sterile surgical gown L	8598	863.36 ± 1.50	7458	748.89 ± 1.31	3160	317.31 ± 0.55	0	0	11,608	1165.62 ± 2.03
Sterile surgical gown M	6736	531.03 ± 0.71	5940	468.28 ± 0.62	4680	368.95 ± 0.49	2727	214.98 ± 0.29	1528	120.46 ± 0.16
Sterile surgical gown S	0	0	0	0	74	5.18 ± 0.01	5472	549.47 ± 0.44	11,608	812.79 ± 0.93
Sterile surgical gown XL	916	105.67 ± 0.63	2056	237.19 ± 1.41	1897	218.85 ± 1.3	143	16.5 ± 0.1	0	0
Sterile surgical gown XXL	1445	185.08 ± 0.48	4064	520.52 ± 1.34	2268	290.49 ± 0.75	1463	187.38 ± 0.48	3085	395.13 ± 1.02
High risk boot cover	225	7.23 ± 0.01	775	24.9 ± 0.02	2300	73.91 ± 0.06	2000	64.27 ± 1.56	1000	32.14 ± 0.78
Boot cover	183,430	648.43 ± 26.60	155,236	548.76 ± 22.51	163,109	576.59 ± 23.65	39,689	140 ± 5.75	47,627	168 ± 6.91
Protective hat	0	0	0	0	90,655	349.48 ± 0.27	153,310	591.01 ± 0.19	75,572	291 ± 0.23
Total	277,127	6702.9 ± 62.64	246,751	6704.86 ± 59.93	402,627	9578.47 ± 69.31	454,429	9632.86 ± 57.9	222,459	7663.45 ± 39.76

**Table 3:** Environmental Footprint characterization of the single-use nonwoven (Part 1).

Impact Category	Unit	Non-Sterile Gown	Patient Sleeveless Gown	Sterile Surgical Gown S	Sterile Surgical Gown M	Sterile Surgical Gown L	Sterile Surgical Gown XL
Climate change	kg CO_2_ eq	0.1426	0.1434	0.2003	0.2221	0.2804	0.3208
		±9.26 × 10^−5^	±4.44 × 10^−4^	±4.58 × 10^−5^	±6.57 × 10^−5^	±1.37 × 10^−4^	±6.11 × 10^−4^
Ozone depletion	kg CFC11 eq	5.96 × 10^−9^	5.99 ×10^−9^	1.16 × 10^−8^	1.25 × 10^−8^	1.49 × 10^−8^	1.66 × 10^−8^
		±1.62 × 10^−19^	±7.75 × 10^−19^	±1.54 × 10^−19^	±2.08 × 10^−19^	±3.87 × 10^−19^	±1.64 × 10^−18^
Ionizing radiation	kBq U-235 eq	2.42 × 10^−3^	2.44 × 10^−3^	4.14 × 10^−3^	4.51 × 10^−3^	5.50 × 10^−3^	6.18 × 10^−3^
		±2.67× 10^−8^	±1.29 × 10^−7^	±1.96 × 10^−8^	±2.71 × 10^−8^	±5.27 × 10^−8^	±2.27 × 10^−7^
Photochemical ozone formation	kg NMVOC eq	5.73× 10^−4^	5.77 × 10^−4^	8.47 × 10^−4^	9.36 × 10^−4^	1.17 × 10^−3^	1.33 × 10^−3^
		±1.49 × 10^−9^	±7.19 × 10^−9^	±8.20 × 10^−10^	±1.17 × 10^−9^	±2.39 × 10^−9^	±1.05 × 10^−8^
Particulate matter	disease inc.	5.89 × 10^−9^	5.93 × 10^−9^	8.62 × 10^−9^	9.52 × 10^−9^	1.19 × 10^−8^	1.36 × 10^−8^
		±1.58 × 10^−19^	±7.59 × 10^−19^	±8.49 × 10^−20^	±1.21 × 10^−19^	±2.47 × 10^−19^	±1.10 × 10^−18^
Human toxicity, non-cancer	CTUh	1.33 × 10^−9^	1.34 × 10^−9^	1.87 × 10^−9^	2.07 × 10^−9^	2.62 × 10^−9^	2.99 × 10^−9^
		±8.05 × 10^−21^	±3.88 × 10^−20^	±4.00 × 10^−21^	±5.71 × 10^−21^	±1.20 × 10^−20^	±5.31 × 10^−20^
Human toxicity, cancer	CTUh	5.30 × 10^−11^	5.33 × 10^−11^	7.66 × 10^−11^	8.47 × 10^−11^	1.06 × 10^−10^	1.21 × 10^−10^
		±1.28 × 10^−23^	±6.13 × 10^−23^	±6.70 × 10^−24^	±9.56 × 10^−24^	±1.96 × 10^−23^	±8.69 × 10^−23^
Acidification	mol H+ eq	6.82 × 10^−4^	6.86 × 10^−4^	1.02 × 10^−3^	1.12 × 10^−3^	1.40 × 10^−3^	1.59 × 10^−3^
		±2.12 × 10^−9^	±1.02 × 10^−8^	±1.19 × 10^−9^	±1.67 × 10^−9^	±3.42 × 10^−9^	±1.50 × 10^−8^
Eutrophication, freshwater	kg P eq	2.69 × 10^−6^	2.71 × 10^−6^	3.84 × 10^−6^	4.24 × 10^−6^	5.35 × 10^−6^	6.11 × 10^−6^
		±3.29 × 10^−14^	±1.59 × 10^−13^	±1.68 × 10^−14^	±2.39 × 10^−14^	±4.99 × 10^−14^	±2.22 × 10^−13^
Eutrophication, marine	kg N eq	1.45 × 10^−4^	1.46 × 10^−4^	2.16 × 10^−4^	2.39 × 10^−4^	2.98 × 10^−4^	3.39 × 10^−4^
		±9.57 × 10^−11^	±4.60 × 10^−10^	±5.33 × 10^−11^	±7.61 × 10^−11^	±1.55 × 10^−10^	±6.82 × 10^−10^
Eutrophication, terrestrial	mol N eq	1.65 × 10^−3^	1.66 × 10^−3^	2.45 × 10^−3^	2.71 × 10^−3^	3.38 × 10^−3^	3.85 × 10^−3^
		±1.24 × 10^−8^	±5.95 × 10^−8^	±6.86 × 10^−9^	±9.78 × 10^−9^	±1.99 × 10^−8^	±8.80 × 10^−8^
Ecotoxicity, freshwater	CTUe	1.51	1.52	2.18	2.42	3.03	3.46
		±1.04 × 10^−2^	±4.99 × 10^−2^	±5.43 × 10^−3^	±7.80 × 10^−3^	±1.60 × 10^−2^	±7.11 × 10^−2^
Land use	Pt	0.51	0.52	0.76	0.84	1.05	1.19
		±1.18 × 10^−3^	±5.84 × 10^−3^	±6.60 × 10^−4^	±9.40 × 10^−4^	±1.92 × 10^−3^	±8.41 × 10^−3^
Water use	m^3^ depriv.	0.0543	0.0546	0.0781	0.0863	0.1085	0.1239
		±1.34 × 10^−5^	±6.44 × 10^−5^	±6.97 × 10^−5^	±9.92 × 10^−6^	±2.05 × 10^−5^	±9.12 × 10^−5^
Resource use, fossils	MJ	4.26	4.28	5.77	6.42	8.16	9.37
		±8.26 × 10^−2^	±3.96 × 10^−1^	±3.80 × 10^−2^	±5.49 × 10^−2^	±1.16 × 10^−1^	±5.21 × 10^−1^

**Table 4:** Environmental Footprint characterization of the single-use nonwoven (Part 2).

Impact Category	Unit	Sterile Surgical Gown XXL	Reinforced Sterile Surgical Gown L	Reinforced Sterile Surgical Gown XL	High Risk Sterile Surgical Gown M	High Risk Sterile Surgical Gown L	High Risk Sterile Surgical Gown XXL
Climate change	kg CO_2_ eq	0.3553	0.5686	0.3993	0.3645	0.4243	0.4962
		±3.25 × 10^−4^	±5.63 × 10^−4^	±7.97 × 10^−4^	±3.66 × 10^−4^	±5.57 × 10^−4^	±3.52 × 10^−3^
Ozone depletion	kg CFC11 eq	1.81 × 10^−8^	1.43 × 10^−6^	2.12× 10^−8^	6.65 × 10^−7^	6.73 × 10^−7^	6.65 × 10^−7^
		±8.44 × 10^−19^	±3.56 × 10^−15^	±2.25 × 10^−18^	±1.22 × 10^−15^	±1.40 × 10^−15^	±6.32 × 10^−15^
Ionizing radiation	kBq U-235 eq	6.77 × 10^−3^	1.17 × 10^−2^	7.18 × 10^−3^	7.53 × 10^−3^	8.56 × 10^−3^	9.75 × 10^−3^
		±1.18 × 10^−7^	±2.39 × 10^−7^	±2.58 × 10^−7^	±1.56 × 10^−7^	±2.27 × 10^−7^	±1.36 × 10^−6^
Photochemical ozone formation	kg NMVOC eq	1.47 × 10^−3^	2.35 × 10^−3^	1.63 × 10^−3^	1.41 × 10^−3^	1.65 × 10^−3^	1.93 × 10^−3^
		±5.57 × 10^−9^	±9.62 × 10^−9^	±1.33 × 10^−8^	±5.47 × 10^−9^	±8.43 × 10^−9^	±5.33 × 10^−8^
Particulate matter	disease inc.	1.50 × 10^−8^	2.54 × 10^−8^	1.65× 10^−8^	1.45 × 10^−8^	1.69 × 10^−8^	1.99 × 10^−8^
		±5.80 × 10^−19^	±1.12 × 10^−18^	±1.36 × 10^−18^	±5.79 × 10^−19^	±8.84 × 10^−19^	±5.66 × 10^−18^
Human toxicity, non-cancer	CTUh	3.32 × 10^−9^	5.54 × 10^−9^	3.68× 10^−9^	3.25 × 10^−9^	3.80 × 10^−9^	4.48 × 10^−9^
		±2.84 × 10^−20^	±5.35 × 10^−20^	±6.77 × 10^−20^	±2.91 × 10^−20^	±4.47 × 10^−20^	±2.87× 10^−19^
Human toxicity, cancer	CTUh	1.34 × 10^−10^	5.03 × 10^−10^	1.50 × 10^−10^	2.21 × 10^−10^	2.44 × 10^−10^	2.70 × 10^−10^
		±4.63 × 10^−23^	±4.41 × 10^−22^	±1.13 × 10^−22^	±1.34 × 10^−22^	±1.84 × 10^−22^	±1.04 × 10^−21^
Acidification	mol H+ eq	1.76 × 10^−3^	2.55 × 10^−3^	1.93 × 10^−3^	1.69 × 10^−3^	1.97 × 10^−3^	2.31 × 10^−3^
		±7.98 × 10^−9^	±1.13 × 10^−8^	±1.86 × 10^−8^	±7.86 × 10^−9^	±1.20 × 10^−8^	±7.63 × 10^−8^
Eutrophication, freshwater	kg P eq	6.76 × 10^−6^	1.88 × 10^−5^	7.26 × 10^−6^	8.54 × 10^−6^	9.68 × 10^−6^	1.10 × 10^−5^
		±1.18 × 10^−13^	±6.16 × 10^−13^	±2.64 × 10^−13^	±2.01 × 10^−13^	±2.90 × 10^−13^	±1.73 × 10^−12^
Eutrophication, marine	kg N eq	3.75 × 10^−4^	5.49 × 10^−4^	4.11 × 10^−4^	3.64 × 10^−4^	4.26 × 10^−4^	4.97 × 10^−4^
		±3.62 × 10^−10^	±5.25 × 10^−10^	±8.45 × 10^−10^	±3.65 × 10^−10^	±5.62 × 10^−10^	±3.53× 10^−9^
Eutrophication, terrestrial	mol N eq	4.26 × 10^−3^	6.62 × 10^−3^	4.65 × 10^−3^	4.07 × 10^−3^	4.76 × 10^−3^	5.58 × 10^−3^
		±4.68 × 10^−8^	±7.64 × 10^−8^	±1.08 × 10^−7^	±4.56 × 10^−8^	±7.02 × 10^−8^	±4.45 × 10^−7^
Ecotoxicity, freshwater	CTUe	3.83	6.7	4.18	3.59	4.22	4.99
		±3.78 × 10^−2^	±7.82 × 10^−2^	±8.74 × 10^−2^	±3.55 × 10^−2^	±5.51 × 10^−2^	±3.56 × 10^−1^
Land use	Pt	1.32	2.26	1.33	1.37	1.58	1.84
		±4.49 × 10^−3^	±8.90 × 10^−3^	±8.85 × 10^−3^	±5.16 × 10^−3^	±7.73 × 10^−3^	±4.84 × 10^−2^
Water use	m^3^ depriv.	0.137	0.3085	0.1595	0.1768	0.1999	0.2268
		±4.84 × 10^−5^	±1.66 × 10^−4^	±1.27 × 10^−4^	±8.60 × 10^−5^	±1.24 × 10^−4^	±7.35 × 10^−4^
Resource use, fossils	MJ	10.39	12.09	11.61	9.59	11.36	13.54
		±2.78 × 10^−1^	±2.55 × 10^−1^	±6.74 × 10^−1^	±2.53 × 10^−1^	±4.00 × 10^−1^	±2.62
Resource use, minerals, and metals	kg Sb eq	7.32 × 10^−6^	−4.50 × 10^−6^	8.33 × 10^−6^	6.73 × 10^−7^	1.85 × 10^−6^	3.47 × 10^−6^
		±1.38 × 10^−13^	±3.53 × 10^−14^	±3.47 × 10^−13^	±1.25 × 10^−15^	±1.06 × 10^−14^	±1.72 × 10^−13^

**Table 5:** Percentual contribution of each material to the carbon footprint of the gowns (≥2%).

Item	Nonwoven PP	Nonwoven Polyester	PET	Fleece	Rubber Bands	Hook-and-Loop	Transportation
Non-sterile gown	93.9	-	-	-	-	-	6.1
Sterile surgical gown size L	85.70	-	-	-	4.79	2.50	6.20
Reinforced sterile surgical gown size L	-	53.80	18.10	20.40	-	-	3.00
High risk sterile surgical gown size L	56.50	-	32.50	-	3.16	-	4.34

## Data Availability

Data employed in this study can be found at https://ecoinvent.org/ (accessed on 21 July 2022).

## Supplementary Materials

The following are available online at https://www.mdpi.com/article/10.3390/polym15051130/s1, Table S1: Nomenclature.
