# Supplementary material for: The Environmental Impacts of Disposable Nonwoven Fabrics during the COVID-19 Pandemic: Case Study on the Francesc de Borja Hospital
Source: Polymers (Basel). 2023 Feb 23;15(5):1130. doi: 10.3390/polym15051130 (PMC10007315; doi:10.3390/polym15051130)
Supplement: Supplementary file 1 [file polymers-15-01130-s001.zip › polymers-2101709-supplementary.pdf]

polymers

Table S1. Nomenclature

Abbreviation	Term
PPE	Personal Protective Equivalent
SMS	spunbond/meltblown/spunbond
AAMI	Association for the Advancement of Medical Instrumentation
AATCC	American Association of Textile Chemists and Colorists
HCW	Health Care Workers
LCA	Life Cycle Assessment
LCI	Life Cycle Inventory
PP	Polypropilene
CO2	Carbon Dioxide
CTUh	Comparative Toxic Unit for human
U-235	Uranium-235
CFC11	Trichlorofluoromethane
MJ	Megajoule
NMVOC	Non-methane volatile organic compound
Sb	Antimony
